# Parameter Estimation of Signal-Dependent Random Noise in CMOS/CCD Image Sensor Based on Numerical Characteristic of Mixed Poisson Noise Samples

**DOI:** 10.3390/s18072276

**Published:** 2018-07-13

**Authors:** Yu Zhang, Guangyi Wang, Jiangtao Xu

**Affiliations:** 1 School of Electronic and Information, Hangzhou Dianzi University, Hangzhou 310018, China; wanggyi@hdu.edu.cn; 2 Key Laboratory for RF Circuits and Systems, Ministry of Education, Hangzhou Dianzi University, Hangzhou 310018, China; 3 School of Microelectronics, Tianjin University, Tianjin 300072, China; xujiangtao@tju.edu.cn

**Keywords:** parameter estimation, signal-dependent random noise, numerical characteristic of mixed Poisson noise samples, complementary metal-oxide semiconductor/charge-coupled device (CMOS/CCD) image sensor

## Abstract

Parameter estimation of Poisson-Gaussian signal-dependent random noise in the complementary metal-oxide semiconductor/charge-coupled device image sensor is a significant step in eliminating noise. The existing estimation algorithms, which are based on finding homogeneous regions, acquire the pair of the variances of noise and the intensities of every homogeneous region to fit the linear or piecewise linear curve and ascertain the noise parameters accordingly. In contrast to the existing algorithms, in this study, the Poisson noise samples of all homogeneous regions in every block image are pieced together to constitute a larger sample following the mixed Poisson noise distribution; then, the mean and variance of the mixed Poisson noise sample are deduced. Next, the mapping function among the noise parameters to be estimated—variance of Poisson-Gaussian noise and that of Gaussian noise corresponding to the stitched region in every block image—is constructed. Finally, the unbiased estimations of noise parameters are calculated from the mapping functions of all the image blocks. The experimental results confirm that the proposed method can obtain lower mean absolute error values of estimated noise parameters than the conventional ones.

## 1. Introduction

In digital imaging systems, images are deteriorated by random noise coming from the complementary metal-oxide semiconductor/charge-coupled device (CMOS/CCD) image sensor [1,2,3,4,5,6,7,8,9,10,11,12,13,14,15,16]. Compared with the additive signal-independent noise model, the signal-dependent noise model is more accurate at characterizing random noise of the CMOS/CCD image sensor [1,2,3,4,5,6,7,8,9,10,11,12]. Most researchers assumed that the signal-dependent noise model of the digital imaging sensor is cond as a Poisson-Gaussian noise model and the validity of the Poisson–Gaussian noise model was certified by CMOS sensors from Nokia camera phones, CCD sensors from Fujifilm cameras and CMOS sensors from Canon cameras [1,2,3,4,5,6,7,8,9,10,11,12]. The Poisson-Gaussian noise model is composed of a signal-dependent term accounting for photon noise (Poisson) and a signal-independent term accounting for the remaining noise in the readout data (Gaussian) [1], as shown in (1).

(1) $$y\left(m,n\right)=x\left(m,n\right)+n_{P-G}\left(x\left(m,n\right)\right)=x\left(m,n\right)+\eta_{p}\left(x\left(m,n\right)\right)+\eta_{g}\left(m,n\right)$$

In (1), *m* and *n* are the horizontal and vertical coordinates of the image pixel, respectively, *y*(*m*,*n*) is the noisy pixel value and *x*(*m*,*n*) is the noiseless value. Further, $\eta_{g}\left(m,n\right)\sim N\left(0,b\right)$ is the zero-mean signal-independent Gaussian noise, $\eta_{p}\left(x\left(m,n\right)\right)$ is the signal-dependent Poisson noise and the mean and variance of $\eta_{p}\left(x\left(m,n\right)\right)$ are equal to ax(m,n). Moreover, *a* is the variance of noise that is associated with analog gain of the CMOS/CCD image sensor and *b* is the variance of noise that is related to readout noise of the CMOS/CCD image sensor [1]. In this study, noise parameters, *a* and *b*, are estimated.

Because the Gaussian noise and Poisson noise are uncorrelated, (2) can be deduced from (1).

(2) $$\sigma_{n_{P-G}}^{2}=ax+b$$

In (2), $\sigma_{n_{P-G}}^{2}$ is the variance of noise $n_{P-G}$.

Parameter estimation is a vital step in achieving a noiseless image. Many algorithms for estimating parameters of the Poisson–Gaussian noise model were presented [1,2,5,6,7]. Owing to the linear relationship between the pixel intensity and the variance of noise, as verified in (2) and the intensity similarity of pixels in the homogeneous region, the main focus of most estimation algorithms is to acquire the parameters of the signal-dependent noise by finding the homogeneous regions [1,5,6]. In [1], analysis and smoothing in wavelets were used to ascertain the local estimation pairs of the pixel intensity and noise variance. Then, the maximum-likelihood approach was employed to fit the global noise model function. In [5], image patches were classified based on their intensity and variance for finding the homogeneous regions that best represent the noise. Then, the weights of the cluster of connected patches were calculated based on the degree of similarity to the noise model. In [6], the true parameter values of Poisson-Gaussian noise were estimated by searching for intersections of the unitary variance contours. Furthermore, methods in other related works did not strive to find the homogeneous regions to address this problem. For example, expectation-maximization was employed to estimate the parameters of Poisson-Gaussian noise in [2]. In [7], the generic Poisson-Gaussian noise model was simplified to a Gaussian-Gaussian noise model and the least squares method was used to estimate the noise model parameters.

The general idea of the existing estimation algorithms based on finding homogeneous regions is to first calculate the noise variance and noiseless intensity of every homogeneous region; then, the pairs of noise variances and noiseless intensities obtained from all homogeneous regions are used to fit the linear or piecewise linear curve; finally, the noise parameters from the fitting curve are acquired.

With consideration of effectiveness to detect and denoising of images, the proposed estimation method is also based on finding homogeneous regions to determine the noise parameters. However, different from the existing estimation algorithms, the proposed algorithm estimates the noise parameters by deriving the mean and variance of the mixed Poisson noise samples that are composed of the Poisson noise of all homogeneous regions in every block image and by building the mapping function among noise parameters to be estimated—variance of Poisson-Gaussian noise and that of Gaussian noise corresponding to the stitched region in every block image.

In this study, the input image is divided into 16 blocks. Then, all homogeneous regions in every block image are detected and denoised in the wavelet domain. Next, all the denoised homogeneous regions are pieced together to form a new stitched image in every block image and histogram analysis is used to obtain the intensities and the corresponding number of every intensity in the stitched noiseless image of every block image. Thus, the mixed Poisson noise samples corresponding to the stitched image in every block image can be obtained according to the definition of the signal-dependent Poisson noise in (1). Next, the mean and variance of the mixed Poisson noise samples in every block image are deduced. The mapping function among noise parameters to be estimated—variance of Poisson-Gaussian noise and that of Gaussian noise corresponding to the stitched region of every block image—is constructed accordingly. Finally, the unbiased estimations of noise parameters are obtained from the mapping functions of 16 block images.

The remainder of the paper is organized as follows. Section 2 presents the proposed algorithm. The experimental results and performance comparison with other state-of-the-art parameter estimation approaches are reported in Section 3. Section 4 concludes our study.

## 2. Proposed Method

**Figure 1:**
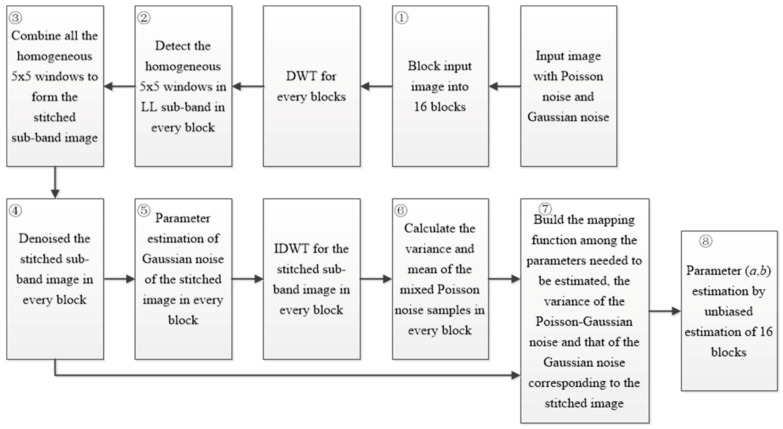
Flowchart of the proposed algorithm.

The flowchart of the proposed method is shown in Figure 1. It is comprised of the following eight main steps.

Step ①: Block the input noisy image. With consideration of computational efficiency, the input noisy image is divided into 16 large blocks.

**Figure 2:**
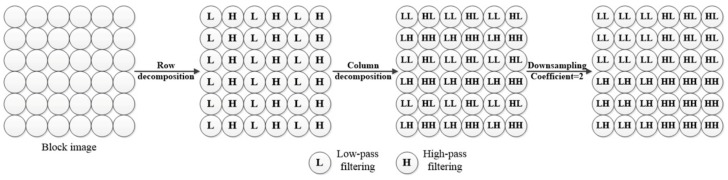
Flowchart of the image wavelet decomposition.

Step ②: Detect the homogeneous regions of every block in the wavelet domain. Owing to the good performance of detecting the homogeneous region in the wavelet domain in [1], each block image is decomposited into four sub-band images *LL*, *HL*, *LH* and *HH*, as shown in Figure 2.

The *LL* sub-band image consists of wavelet coefficients that are obtained by performing low-pass wavelet filtering in the row and column of the block image; the *HL* sub-band image consists of wavelet coefficients that are obtained by performing high-pass wavelet filtering in the row and low-pass wavelet filtering in the column of the block image; the *LH* sub-band image consists of wavelet coefficients that are obtained by performing low-pass wavelet filtering in the row and high-pass wavelet filtering in the column of the block image; and the *HH* sub-band image consists of wavelet coefficients that are obtained by performing high-pass wavelet filtering in the row and column of the block image. With respect to the orthogonality and regularity, the Daubechies wavelet basis (db6) is employed in this study. With consideration of complexity and accuracy of computation, the standard deviation of the 5 × 5 slipping window in the *LL* sub-band is calculated and compared with the threshold shown in (3) to ascertain the homogeneous region.

(3) $$\left\{ \begin{array}{l} \mu_{LL}=\frac{1}{25}\sum_{i=1}^{5}\sum_{j=1}^{5}W\left\{LL\right\}\left(i,j\right)\\ SD_{LL}=\sqrt{\frac{\sum_{i=1}^{5}\sum_{j=1}^{5}{\left(W\left\{LL\right\}\left(i,j\right)-\mu_{LL}\right)}^{2}}{25}}<\delta \end{array}\right.$$

In (3), *W*{*LL*} is the wavelet coefficient of the 5 × 5 slipping window in the *LL* sub-band, $\mu_{LL}$ is the mean of the 5 × 5 slipping window in the *LL* sub-band, $SD_{LL}$ denotes the standard deviation of the 5 × 5 slipping window in the *LL* sub-band and *δ* represents the threshold of $SD_{LL}$. If the standard deviation of the 5 × 5 slipping window is less than *δ*, this region is homogeneous and the coordinates of the central wavelet coefficient in the 5 × 5 slipping window are recorded.

**Figure 3:**
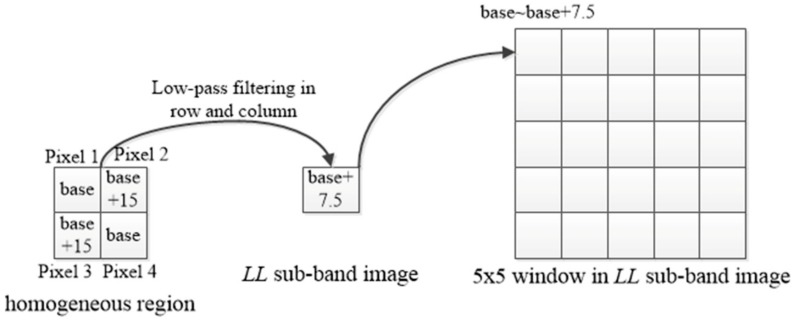
Setting method of threshold.

Furthermore, the pixel intensities in the homogeneous region are close and the maximal intensity difference of two pixels in the homogeneous region can be set to 15, according to the literature [17]. The setting of threshold *δ* of $SD_{LL}$ in (3) is explained in Figure 3. The base value of the homogeneous region is the minimal value of this homogeneous region. Because the *LL* sub-band image is obtained by performing low-pass filtering between adjacent pixels in the row and column, the range of *LL* wavelet coefficient corresponding to the homogeneous region is from base value corresponding to this homogenous region to base value +7.5. That is, the value range of all the *LL* wavelet coefficients in the 5 × 5 window is from the base value of this homogeneous region to base value +7.5. Through calculation, the value of $SD_{LL}$ in the 5 × 5 window will reach its maximum when there are 13 *LL* wavelet coefficients equal to base value +7.5 and the remaining 12 *LL* wavelet coefficients equal to base value. As a result, *δ* in (3) is set to the maximum of $SD_{LL}$, that is, *δ* = 3.75.

**Figure 4:**
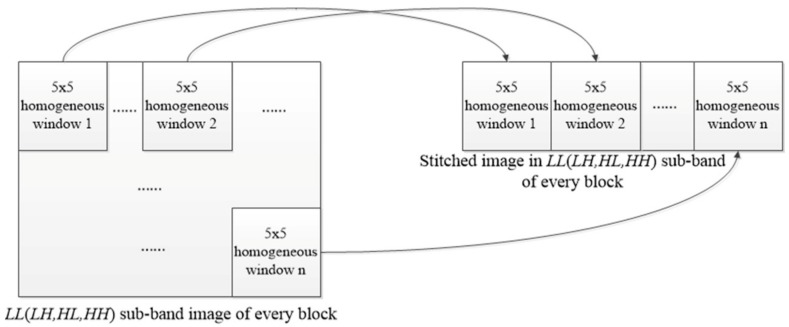
Extracting all homogeneous 5 × 5 windows to form the stitched sub-band image.

Step ③ Combine all homogeneous 5 × 5 windows to form the stitched sub-band image. According to the coordinates of the central wavelet coefficient in the 5 × 5 slipping window, all homogeneous windows in the *LL* sub-band of each block are extracted and stitched to form a new stitched image. The same operation will be performed for *LH*, *HL* and *HH* sub-bands and the stitched images in *LH*, *HL and HH* sub-bands can be obtained accordingly. The extraction and combination process is depicted in Figure 4. The stitched wavelet coefficients in *LL*, *LH*, *HL* and *HH* wavelet sub-band can be reconstructed as a stitched image in every block, denoted as *y_Si_*, *i =* 1~16.

Step ④ Parameter estimation of Gaussian noise of the stitched image in every block. The median absolute deviation (MAD) is used to estimate the standard deviation of Gaussian noise of the stitched image *y_Si_* [7] as shown in (4).

(4) $${\hat{\sigma }}_{g-S_{i}}=\frac{Median\left(\left|W\left\{HH\right\}\right|\right)}{0.6745},i=1\sim 16$$

In (4), ${\hat{\sigma }}_{g-S_{i}}$ (*i =* 1~16) is the estimated standard deviation of the Gaussian noise of the stitched image *y_Si_* in every block, *Median*(●) is the MAD value and W{HH} is the *HH* sub-band wavelet coefficient of the stitched image in every block shown in Figure 4. It can be determined from (1) that $b_{i}={\hat{\sigma }}_{g-S_{i}}^{2}$, *i =* 1~16.

Step ⑤ Denoise the stitched sub-band image of every block. In order to obtain the denoised stitched sub-band image, the mean values of the 5 × 5 slipping window in *LH*, *HL* and *HH* sub-bands—which are located in the corresponding position as the 5 × 5 slipping window in the *LL* sub-band—are calculated as (5). Then, all the wavelet coefficients of the 5 × 5 slipping window in *LH*, *HL* and *HH* sub-bands are replaced by these three mean values.

(5) $$\mu_{\left\{LH,HL,HH\right\}}=\frac{1}{25}\sum_{i=1}^{5}\sum_{j=1}^{5}W\left\{LH,HL,HH\right\}\left(i,j\right)$$

In (5), $\mu_{\left\{LH,HL,HH\right\}}$ are the mean values of the 5 × 5 slipping window in the *LH*, *HL* and *HH* sub-bands and W{LH,HL,HH} are the wavelet coefficients of the 5 × 5 slipping window in *LH*, *HL* and *HH* sub-bands of the noisy image.

After all the homogeneous regions are denoised, the block image in the wavelet domain is translated into a temporal image, denoted as *S_i_*, *i =* 1~16.

**Figure 5:**
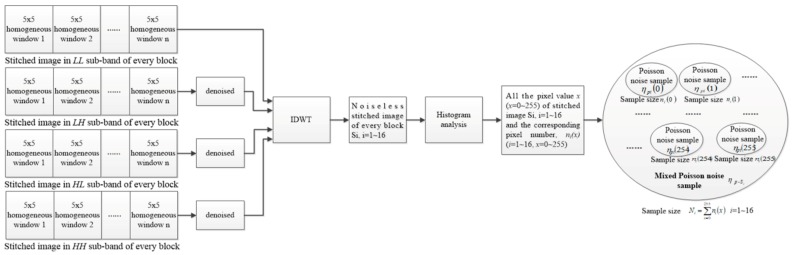
Forming process of the mixed Poisson noise $\eta_{p-S_{i}}$.

Step ⑥ Calculate the variance and mean of the mixed Poisson noise samples. According to (1), the Poisson-Gaussian noise corresponding to the noiseless stitched region *S_i_* is denoted as $n_{P-G-S_{i}}$ (*i =* 1~16); the mixed Poisson noise corresponding to the noiseless stitched region *S_i_* is denoted as $\eta_{p-S_{i}}$ (*i =* 1~16); and the Gaussian noise corresponding to the noiseless stitched region *S_i_* is denoted as $\eta_{g-S_{i}}$ (*i =* 1~16). The forming process of the mixed Poisson noise $\eta_{p-S_{i}}$ (*i =* 1~16) corresponding to the noiseless stitched image *S_i_* is shown in Figure 5.

It can be seen from Figure 5 that the histogram analysis offers the pixel value *x* (*x =* 0~255) and the corresponding pixel number (denoted as *n_i_(x)* (*i =* 1~16, *x =* 0~255)) of every noiseless stitched image (denoted as *S_i_* (*i =* 1~16)). Because the parasitic Poisson noise is signal-dependent and $\eta_{p}\left(x\right)\sim P\left(ax\right)$, as shown in (1), the Poisson noise samples $\eta_{pi}\left(x\right)$ (*i =* 1~16, *x =* 0~255) and the corresponding sample size *n_i_(x)* (*i =* 1~16, *x =* 0~255) can be obtained according to histogram analysis. All the noise samples $\eta_{pi}\left(x\right)$ constitute the mixed Poisson noise sample $\eta_{p-S_{i}}=\left\{\eta_{pi}\left(0\right),\eta_{pi}\left(1\right),\eta_{pi}\left(2\right)\ldots \ldots \eta_{pi}\left(255\right)\right\}$ (*i =* 1~16) corresponding to *S_i_* (*i =* 1~16) and the sample size of $\eta_{p-S_{i}}$ can be represented as *N_i_* (*i =* 1~16). As a result, $N_{i}=\sum_{x=0}^{255}n_{i}\left(x\right)$ (*i =* 1~16) and the unbiased estimations of the mean of $\eta_{p-S_{i}}$ (*i =* 1~16) can be obtained as (6).

(6) $${\hat{\mu }}_{p-S_{i}}=\frac{\sum_{k=1}^{N_{i}}\eta_{p-S_{i}}\left(k\right)}{N_{i}}=\frac{\sum_{x=0}^{255}\sum_{j=1}^{n_{i}\left(x\right)}\eta_{pi}\left(x,j\right)}{N_{i}}=\frac{\sum_{x=0}^{255}n_{i}\left(x\right){\hat{\mu }}_{pi}\left(x\right)}{N_{i}}=\sum_{x=0}^{255}\frac{n_{i}\left(x\right)}{N_{i}}\left(a_{i}x\right)$$

In (6), ${\hat{\mu }}_{p-S_{i}}$ is the unbiased estimation of the mean of the mixed Poisson noise sample $\eta_{p-S_{i}}$ and ${\hat{\mu }}_{pi}\left(x\right)$ is the unbiased estimation of mean of Poisson noise samples $\eta_{pi}\left(x\right)$.

The unbiased estimations of the variance of $\eta_{p-S_{i}}$ (*i =* 1~16) can be obtained as (7).

(7) $${\hat{\sigma }}_{p-S_{i}}^{2}=\frac{1}{\left(N_{i}-1\right)}\sum_{k=1}^{N_{i}}{\left(\eta_{p-S_{i}}\left(k\right)-{\hat{\mu }}_{p-S_{i}}\right)}^{2}$$

Substituting ${\hat{\mu }}_{p-S_{i}}$ with (6), the unbiased estimations of the variance of $\eta_{p-S_{i}}$ can be obtained as (8).

(8) $$\begin{array}{l} {\hat{\sigma }}_{p-S_{i}}^{2}=\frac{1}{\left(N_{i}-1\right)}\sum_{k=1}^{N_{i}}{\left(\eta_{p-S_{i}}\left(k\right)-{\hat{\mu }}_{p-S_{i}}\right)}^{2}\\ =\frac{1}{\left(N_{i}-1\right)}\sum_{k=1}^{N_{i}}{\left(\eta_{p-S_{i}}(k)-\frac{\sum_{x=0}^{255}n_{i}\left(x\right){\hat{\mu }}_{pi}(x)}{N_{i}}\right)}^{2}\\ =\frac{1}{\left(N_{i}-1\right)}[\sum_{X=0}^{255}\sum_{J=1}^{N_{i}(x)}{\left(\eta_{pi}(x,j)-\frac{\sum_{x=0}^{255}n_{i}\left(x\right){\hat{\mu }}_{pi}(x)}{N_{i}}\right)}^{2}]\\ =\frac{1}{\left(N_{i}-1\right)}[\sum_{X=0}^{255}\sum_{J=1}^{N_{i}(x)}{\left(\eta_{pi}(x,j)-{\hat{\mu }}_{pi}(x)-\frac{\sum_{x=0}^{255}n_{i}\left(x\right){\hat{\mu }}_{pi}(x)}{N_{i}}+{\hat{\mu }}_{pi}(x)\right)}^{2}]\\ =\frac{1}{\left(N_{i}-1\right)}[\sum_{X=0}^{255}(n_{i})(x)-1){\hat{\sigma }}_{pi}^{2}(x)+\frac{1}{N_{i}}(\sum_{X=0}^{254}\sum_{k=x+1}^{255}n_{i}(x)n_{i}(k){\left({\hat{\mu }}_{pi}(x)-{\hat{\mu }}_{pi}(k)\right)}^{2})]\\ =\frac{1}{\left(N_{i}-1\right)}[\sum_{X=0}^{255}(n_{i})(x)-1)(a_{i}x)+\frac{1}{N_{i}}(\sum_{X=0}^{254}\sum_{k=x+1}^{255}n_{i}(x)n_{i}(k){\left(a_{i}x-a_{i}k\right)}^{2})]i=1\sim 16 \end{array}$$

In (8), ${\hat{\sigma }}_{p-S_{i}}^{2}$ is the unbiased estimation of variance of the mixed Poisson noise sample $\eta_{p-S_{i}}$ and ${\hat{\sigma }}_{pi}^{2}\left(x\right)$ is the unbiased estimation of the variance of Poisson noise samples $\eta_{pi}\left(x\right)$.

Step ⑦ Build the mapping function among the noise parameters to be estimated, variance of $n_{P-G-S_{i}}$ and variance of $\eta_{g-S_{i}}$. $n_{P-G-S_{i}}$ can be obtained by calculating the difference between the noisy stitched image *y_Si_* and the noiseless stitched image *S_i_*, *i =* 1~16. The unbiased estimation of variance of $n_{P-G-S_{i}}$ (*i =* 1~16) can be calculated by (9).

(9) $$\left\{ \begin{array}{l} {\hat{\mu }}_{n_{P-G-S_{i}}}=\frac{\sum_{k=1}^{N_{i}}n_{P-G-S_{i}}\left(k\right)}{N_{i}}\\ {\hat{\sigma }}_{n_{P-G-S_{i}}}^{2}=\frac{1}{\left(N_{i}-1\right)}\sum_{k=1}^{N_{i}}{\left(n_{P-G-S_{i}}\left(k\right)-{\hat{\mu }}_{n_{P-G-S_{i}}}\right)}^{2} \end{array}\right.,i=1\sim 16$$

In (9), ${\hat{\mu }}_{n_{P-G-S_{i}}}$ is the unbiased estimation of the mean of $n_{P-G-S_{i}}$ and ${\hat{\sigma }}_{n_{P-G-S_{i}}}^{2}$ denotes the unbiased estimation of the variance of $n_{P-G-S_{i}}$.

As the Poisson noise and Gaussian noise are irrelevant, the variance of $n_{P-G-S_{i}}$ is the sum of the variance of $\eta_{p-S_{i}}$ and the variance of $\eta_{g-S_{i}}$, as shown in (10).

(10) $${\hat{\sigma }}_{n_{P-G-S_{i}}}^{2}={\hat{\sigma }}_{p-S_{i}}^{2}+{\hat{\sigma }}_{g-S_{i}}^{2}$$

From (7) to (10), let $L_{i}=\frac{\sum_{x=0}^{255}\left(n_{i}\left(x\right)-1\right)x}{N_{i}-1}$ and $M_{i}=\frac{\sum_{x=0}^{254}\sum_{k=x+1}^{255}n_{i}\left(x\right)n_{i}\left(k\right){\left(x-k\right)}^{2}}{N_{i}\left(N_{i}-1\right)}$. Thus, (11) can be obtained.

(11) $$a_{i}L_{i}+a_{i}{}^{2}M_{i}={\hat{\sigma }}_{n_{P-G-S_{i}}}^{2}-{\hat{\sigma }}_{g-S_{i}}^{2},i=1\sim 16$$

As a result, the estimation of parameter *a_i_* in every block can be acquired from (12).

(12) $$a_{i}=\frac{-L_{i}\pm \sqrt{{\left(L_{i}\right)}^{2}-4\times M_{i}\times \left({\hat{\sigma }}_{g-S_{i}}^{2}-{\hat{\sigma }}_{n_{P-G-S_{i}}}^{2}\right)}}{2M_{i}},i=1\sim 16$$

In (12), the values of *a_i_* (*i =* 1~16) are positive.

Step ⑧ Perform unbiased parameter estimation of 16 block images. From (7) and (12), the parameters *a* and *b* in each block image are acquired. To improve the estimation accuracy, the unbiased estimations of *a* and *b* in 16 blocks are calculated as (13).

(13) $$\hat{a}=\frac{\sum_{i=1}^{16}a_{i}}{16},\;\hat{b}=\frac{\sum_{i=1}^{16}{\hat{\sigma }}_{g-S_{i}}^{2}}{16}$$

## 3. Simulation Results and Comparison

The proposed method is compared with the parameter estimation methods in [1,5] that are based on finding the homogeneous region. In addition, the proposed method is compared with the parameter estimation method in [7]; the latter method is not based on finding the homogeneous region but it has good performance in estimating the noise parameter.

### 3.1. Simulation and Comparison Results with Kodak Test Image

**Figure 6:**
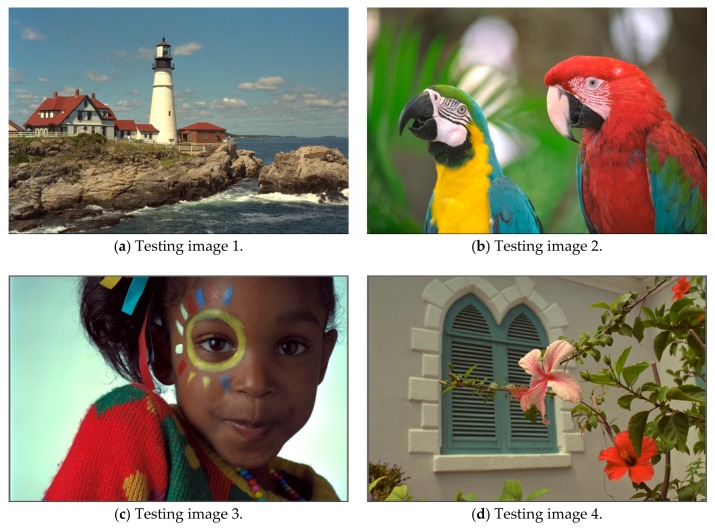

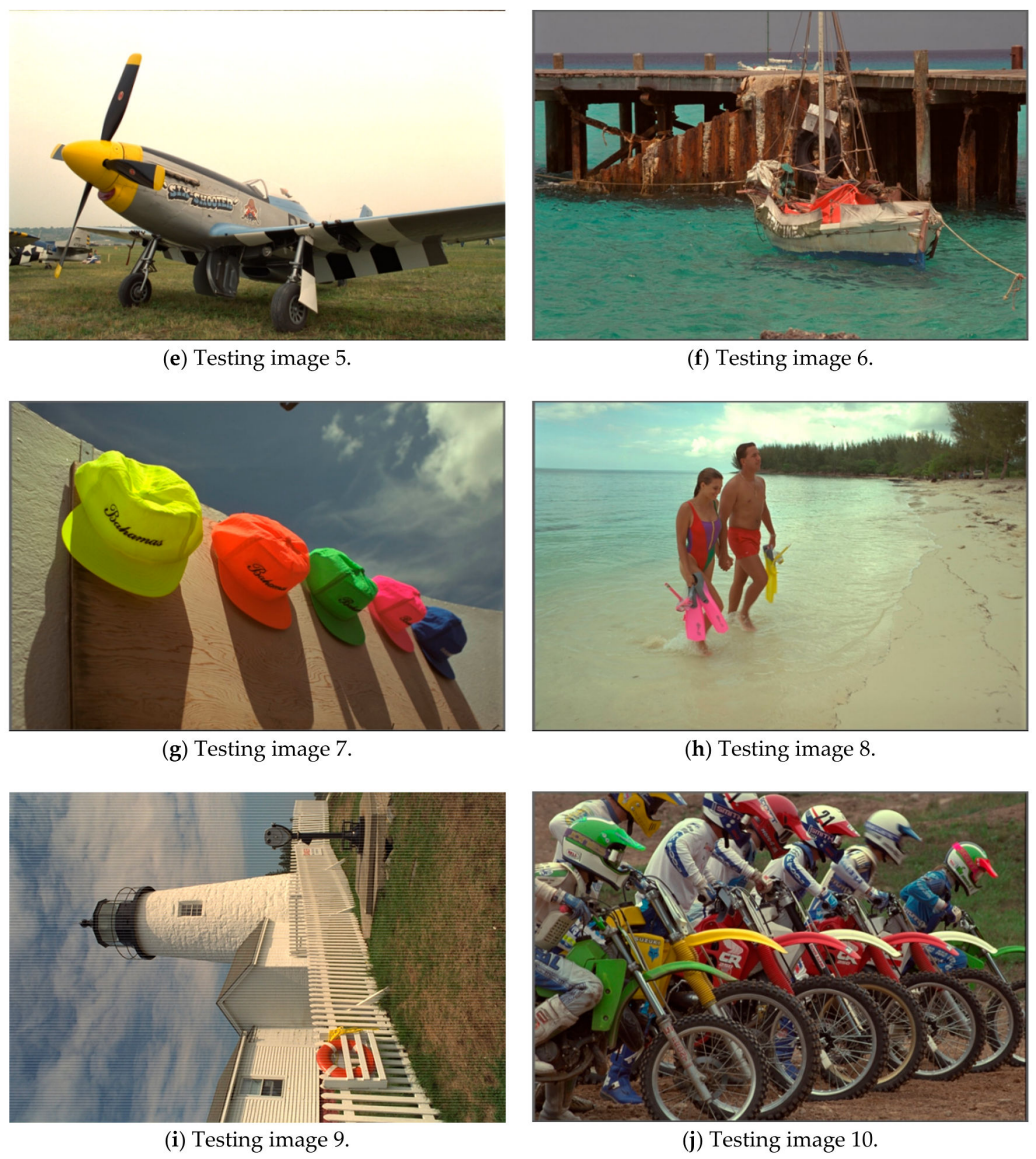
Ten Kodak standard noiseless testing images.

In order to evaluate different estimation methods fairly and objectively, ten 512 × 768 noiseless and standard test images supplied by Kodak company, as shown in Figure 6, are employed as testing images. The sets of noise parameters, *a =* {0.005, 0.010, 0.015} and *b =* {0.016, 0.036, 0.064}, are placed into the R, G, B channels of ten testing images respectively, to construct the noisy images.

**Figure 7:**
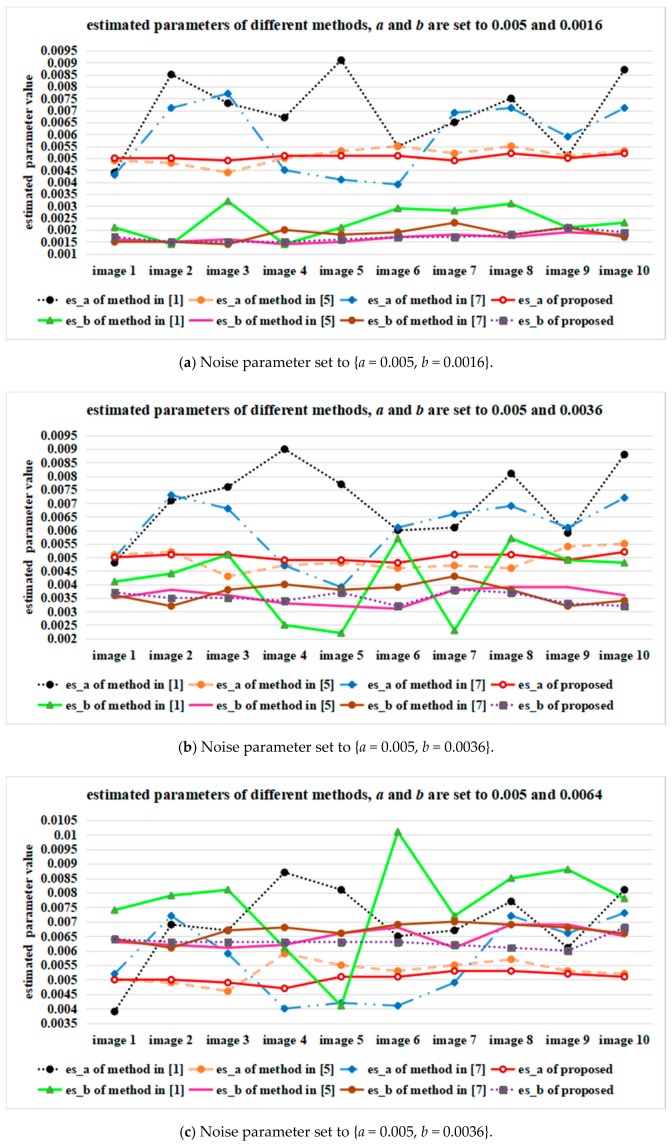

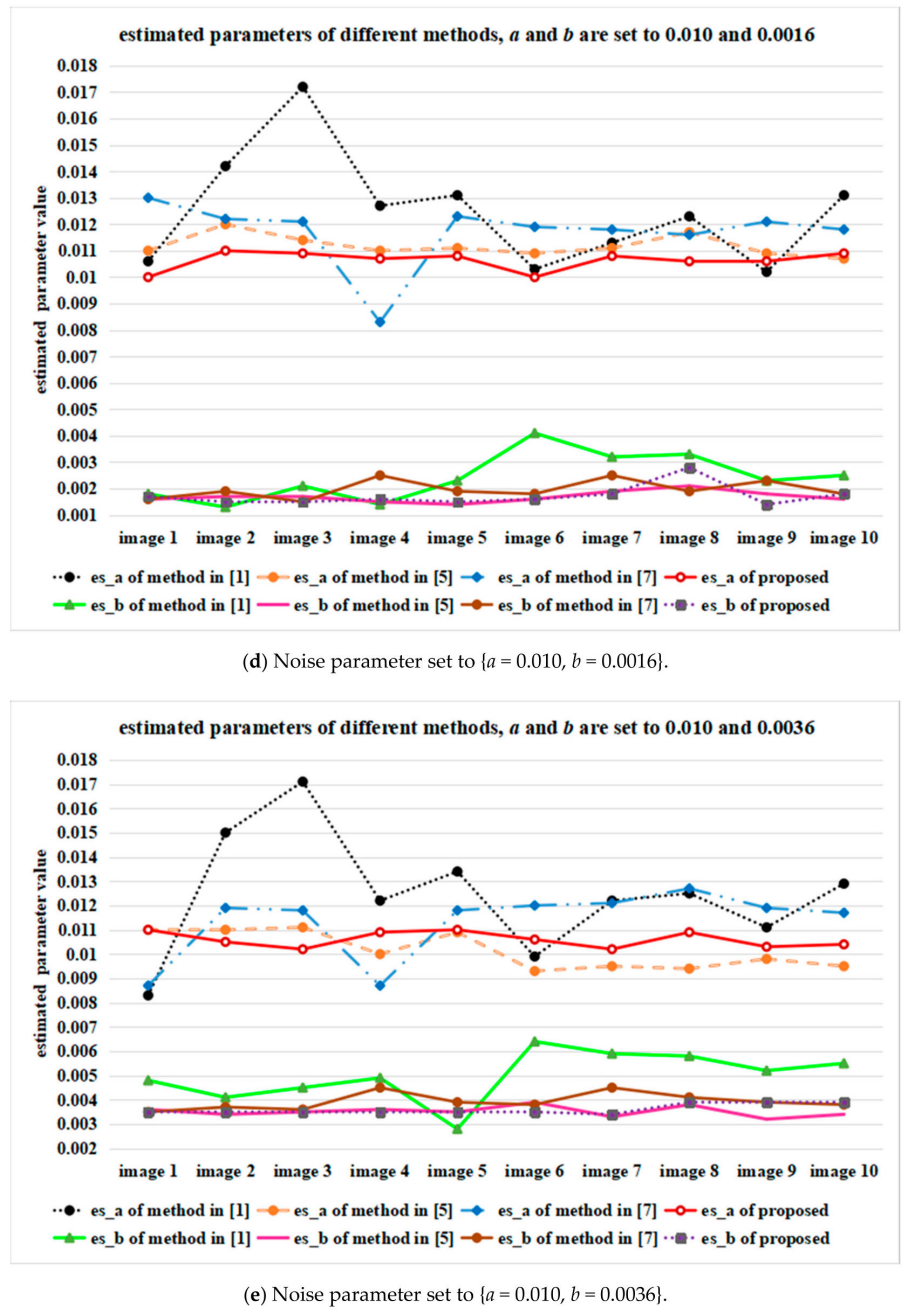

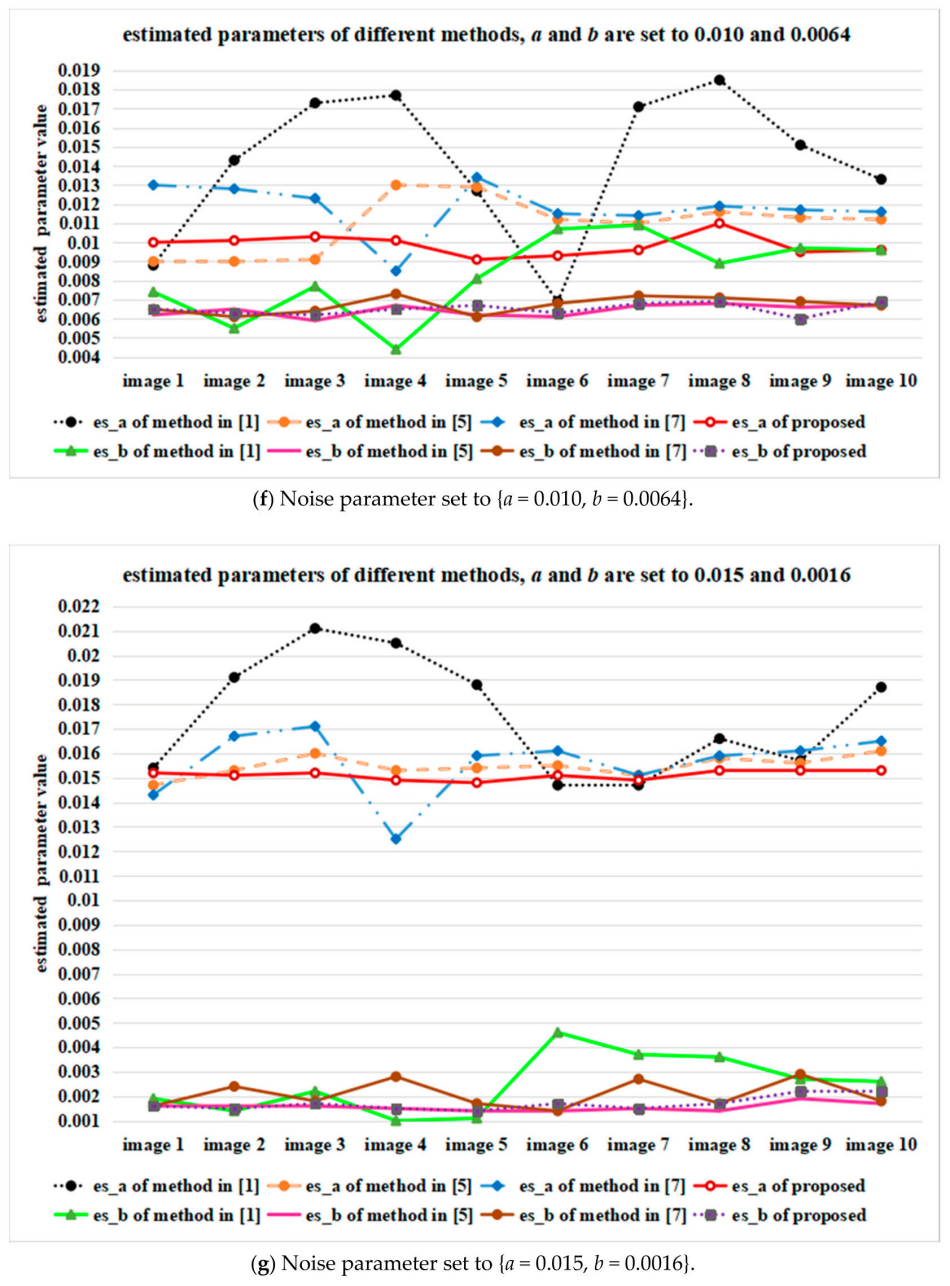

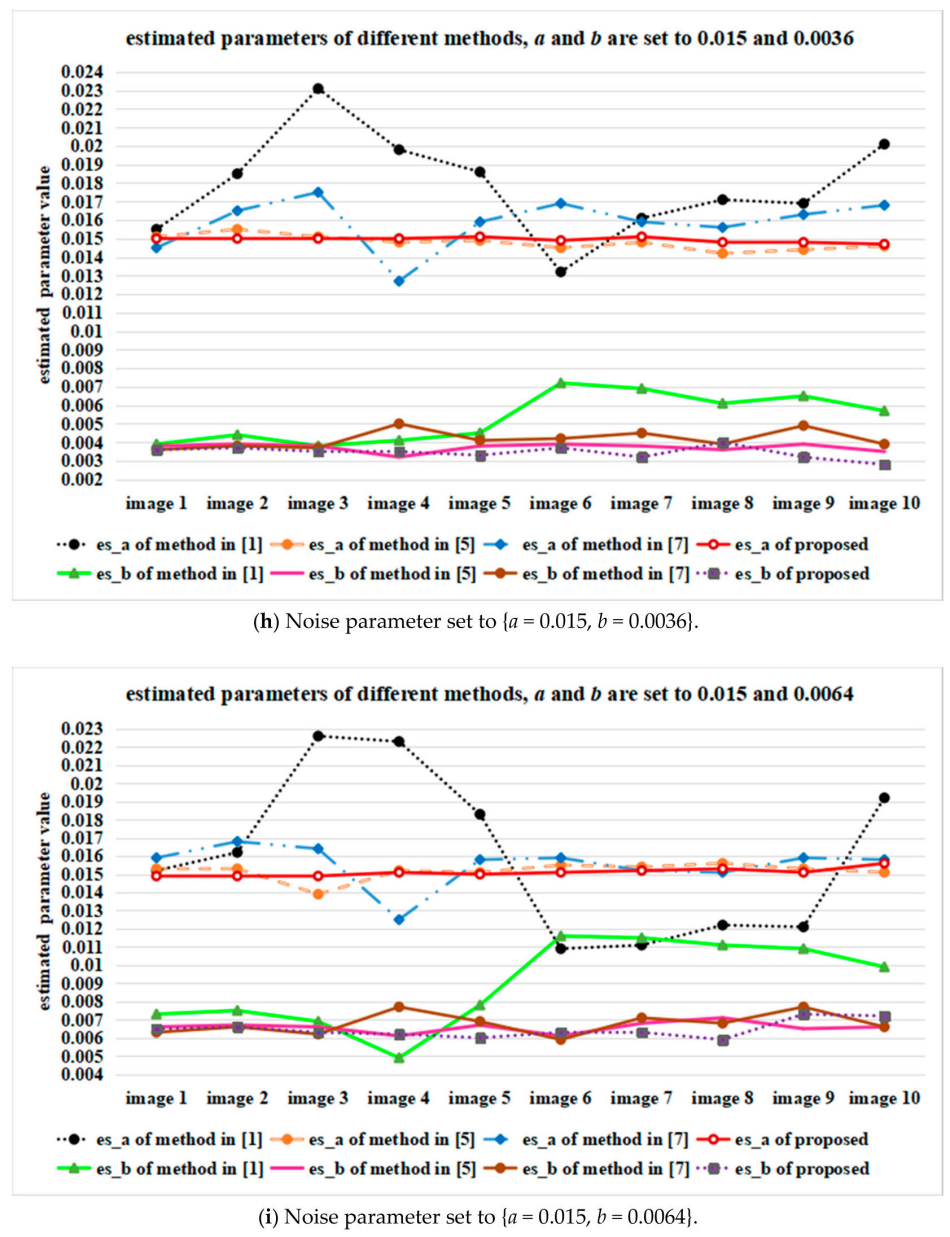
The average values of estimated noise parameters in R, G, B channels of the ten Kodak testing images, by using different estimation methods.

**Figure 8:**
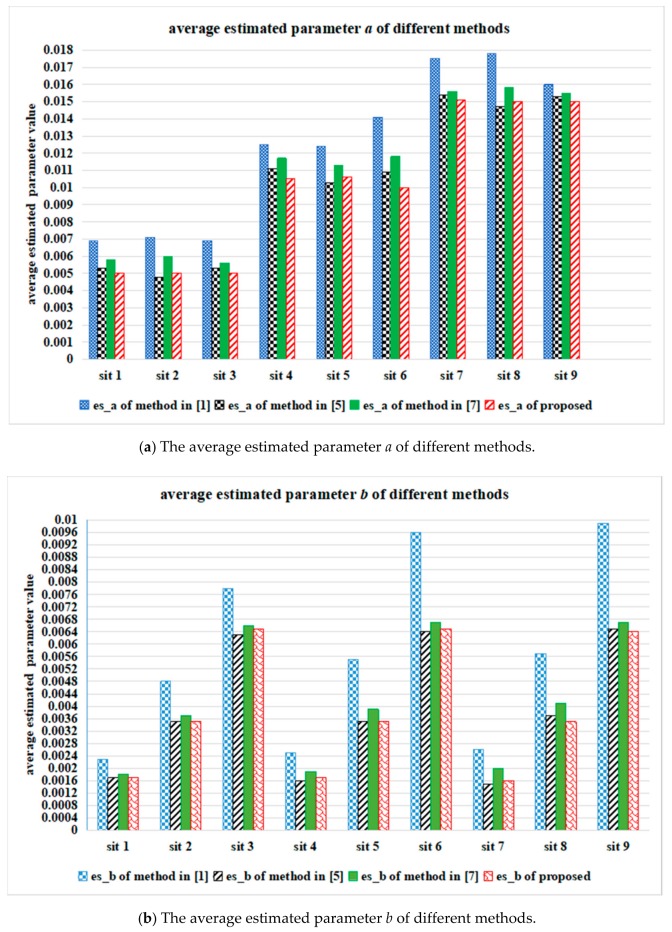
The average values of estimated noise parameters of the ten Kodak testing images, by using different estimation methods.

Figure 7 shows the average values of estimated signal-dependent noise parameters *a* and *b* in R, G, B channels of the ten Kodak testing images, which were processed by different parameter estimation methods. In Figure 7, es_a is the estimated value of parameter *a* and es_b is the estimated value of parameter *b*. Figure 8 presents the average estimation values of ten testing images processed by different parameter estimation methods, where sit 1 means {*a =* 0.005 and *b =* 0.0016}, sit 2 means {*a =* 0.005 and *b =* 0.0036}, sit 3 means {*a =* 0.005 and *b =* 0.0064}, sit 4 means {*a =* 0.01 and *b =* 0.0016}, sit 5 means {*a =* 0.01 and *b =* 0.0036}, sit 6 means {*a =* 0.01 and *b =* 0.0064}, sit 7 means {*a =* 0.015 and *b =* 0.0016}, sit 8 means {*a =* 0.015 and *b =* 0.0036}, sit 9 means {*a =* 0.015 and *b =* 0.0064}.

Figure 7 and Figure 8 clearly show that the estimated value of parameter *a* processed by the proposed method is closer to the preset parameter than that processed by other methods, even when the Poisson noise component is strong. On the contrary, the proposed method obtains comparable results to those of the other methods when the Gaussian noise component is strong.

To assess the complexity of different estimation methods, the running times of Matlab 7.1 implementations to process the Kodak testing images are listed (*a =* 0.015 and *b =* 0.0064). The hardware simulation environments consisted of an Intel Core™ 2 1.8 GHz CPU and 1 GB RAM. The average running times of the green channel of ten testing images are given as follows: that of the method in [1] was 12 s; the method in [5] was 5 s; that in [7] was 4 s; and that of the proposed method was 2 s. It should be emphasized that this comparison only serves as a reference; the running times also heavily depend on the optimization of the program codes. It is observed that the proposed method is faster than other methods for the given noise parameters.

### 3.2. Simulation and Comparison Results with Actual Image of CMOS Image Sensor

**Figure 9:**
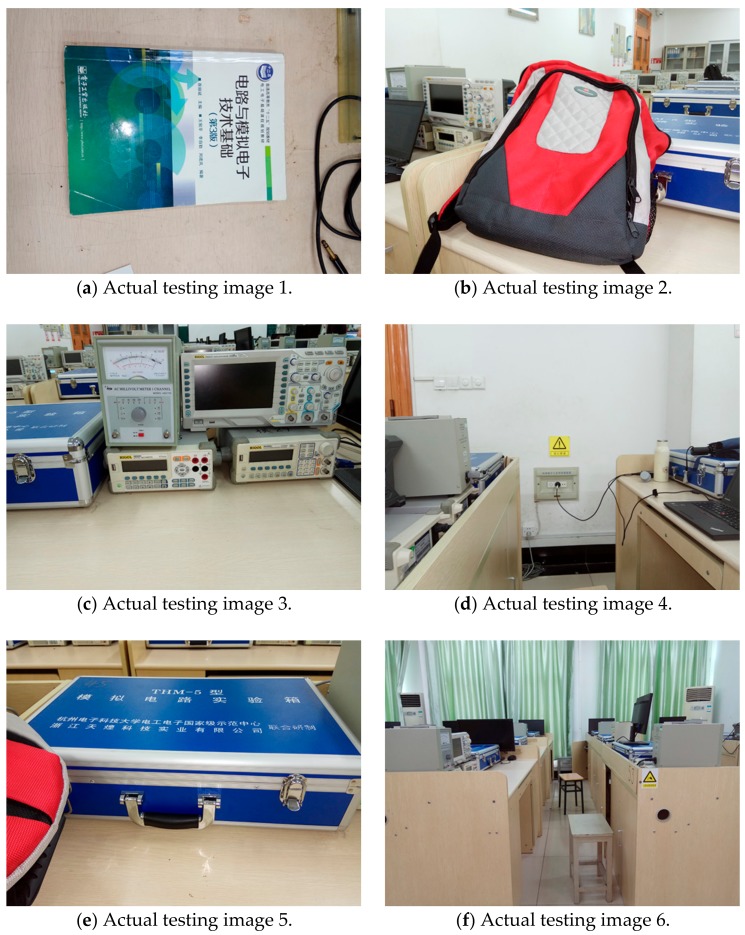
The actual testing images captured from CMOS image sensor.

The aforementioned parameter estimation methods are assessed with the actual 640 × 480 images of CMOS image sensor in Figure 9. Every testing image in Figure 9 is taken by ov5640 CMOS image sensor 50 times to the same static scene; then, the 50 static images are averaged to form the noiseless database. Next, the sets of noise parameters, *a =* {0.005, 0.010, 0.015} and *b =* {0.016, 0.036, 0.064}, are placed into the R, G, B channels of six testing images to construct the noisy images.

**Figure 10:**
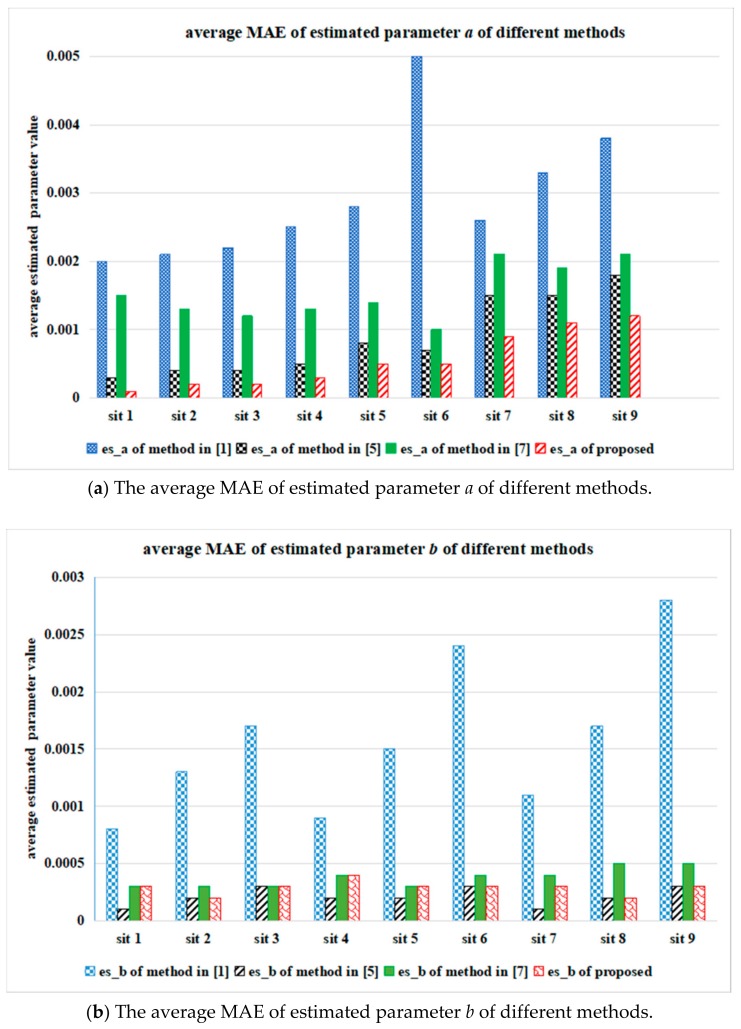
The average MAE values of estimated noise parameters of the six actual testing images from CMOS image sensor, by using different estimation methods.

Figure 10 shows the average mean absolute error (*MAE*) values of estimated signal-dependent noise parameters *a* and *b* of the R, G, B channels of the six testing images. The meanings of sit 1-sit 9 in Figure 10 are similar to those in Figure 8. The *MAE* values of *a* and *b* can be calculated by (14). The smaller the *MAE* value is, the closer the estimated parameter value is to the preset noise parameter value.

(14) $$\left\{ \begin{array}{l} MAE\_a=\frac{1}{10}\sum_{i=1}^{10}\left|{\hat{a}}_{i}-a\right|\\ MAE\_b=\frac{1}{10}\sum_{i=1}^{10}\left|{\hat{b}}_{i}-b\right| \end{array}\right.$$

In (14), *MAE_a* and *MAE_b* are the *MAE* values of noise parameters *a* and *b*, respectively. In (14), *a* and *b* are the given noise parameters and $\hat{a}$ and $\hat{b}$ are the estimated noise parameters of the green channel of the ten testing images.

It can be seen from Figure 10 that the proposed method stably outperforms the other methods even when the Poisson noise component is strong; further, the proposed method obtains results comparable to those of the other methods when the Gaussian noise component is strong. A similar conclusion can be derived from Figure 7, Figure 8 and Figure 10 that the proposed estimation method can steadily obtain a more accurate estimated value of the Poisson noise parameter than other methods. This is because parameter estimation is based on the numerical characteristics of mixed Poisson noise samples.

## 4. Conclusions

In this paper, a parameter estimation method of signal-dependent random noise based on the numerical characteristics of mixed Poisson noise samples was proposed. By deducing the mean and variance of the mixed Poisson noise samples corresponding to the stitched region and building the mapping function among the parameters to be estimated—variance of the Poisson-Gaussian noise and that of Gaussian noise corresponding to the stitched region in every block image—the noise parameters were estimated. The experimental results indicated that the proposed method achieved lower MAE values of the noise parameter and lower computational complexity than the existing algorithms.
